# Effect of pain on deafferentation-induced modulation of somatosensory evoked potentials

**DOI:** 10.1371/journal.pone.0206141

**Published:** 2018-10-22

**Authors:** Jean-Daniel Dubois, Isabelle Poitras, Julien I. A. Voisin, Catherine Mercier

**Affiliations:** 1 Center for Interdisciplinary Research in Rehabilitation and Social Integration, Quebec City, Québec, Canada; 2 Department of Rehabilitation, Laval University, Pavillon Ferdinand-Vandry, Quebec City, Québec, Canada; University of Rome, ITALY

## Abstract

There is a large body of evidence showing substantial sensorimotor reorganizations after an amputation. These reorganizations are believed to contribute to the development of phantom limb pain, but alternatively, pain might influence the plasticity triggered by the deafferentation. The aim of this study was to test whether pain impacts on deafferentation-induced plasticity in the somatosensory pathways. Fifteen healthy subjects participated in 2 experimental sessions (Pain, No Pain) in which somatosensory evoked potentials (SSEPs) associated with electrical stimulation of the ulnar nerve were assessed before and after temporary ischemic deafferentation induced by inflation of a cuff around the wrist. In the *Pain* session capsaicin cream was applied on the dorsum of the hand 30 minutes prior to cuff inflation. Results show that pain decreased the amplitude of the N20 (main effect of condition, p = 0.033), with a similar trend for the P25. Temporary ischemic deafferentation had a significant effect on SSEPs (main effect of time), with an increase in the P25 (p = 0.013) and the P45 amplitude (p = 0.005), together with a reduction of the P90 amplitude (p = 0.002). Finally, a significant time x condition interaction, reflecting state-dependent plasticity, was found for the P90 only, the presence of pain decreasing the reduction of amplitude observed in response to deafferentation. In conclusion, these results show that nociceptive input can influence the plasticity induced by a deafferentation, which could be a contributing factor in the cortical somatosensory reorganization observed in chronic pain populations.

## Introduction

Most amputees experience a phantom limb sensation, which is the vivid perception that their missing limb is still there [1]. Unfortunately, many of them also experience phantom limb pain (PLP), i.e. pain perceived as arising from their missing limb, although the estimated prevalence varies from 29% to 72% across studies [2–5]. Some clinical studies have provided evidence that the presence of pain in a limb prior to its amputation is a risk factor for the development of PLP [3, 5–8] (but see [4] and [9] for contrasting results). Furthermore, several amputees report pain in their phantom limb that is qualitatively similar to the pain felt just before amputation [8, 10, 11], and this led to a model based on somatosensory pain memories [12]. These clinical observations in amputees linking pre-amputation pain with PLP are in line with observations in animal studies showing that nociceptive stimuli applied just prior to deafferentation enhance the development of autotomy (self-mutilation behavior believed to reflect pain) after deafferentation [13–15]. Similarly, clinical studies suggest that it is the presence of pain at or close to the moment of the amputation, rather than the length of time that a patient’s limb has been painful before the amputation, that is related to the presence of pain after amputation [7, 16].

Based on these observations, it has been hypothesized that the presence of pain might have a modulatory effect on the plasticity that will be induced by the lesion [17]. There is a large body of evidence showing that substantial cortical and subcortical reorganization occurs in the sensorimotor system after an amputation [18–21]. These reorganizations, characterized by a degradation of the missing hand representation and remapping of other body part representations, are proposed to cause or contribute to PLP [22–27]. This remains controversial however, as several studies did not find significant associations between the extent of reorganization and PLP, and as the cross-sectional designs generally used in clinical studies make it difficult to disentangle the cause from the consequence [28–34]. Recently, we used an experimental model of amputation (temporary ischemic deafferentation) in healthy controls to assess the effect of “pre-amputation” pain on the changes induced in corticospinal excitability, in response to the deafferentation. We showed that the presence of hand pain itself does not impact on corticospinal excitability of forearm muscles located above the ischemic block, but that it does enhance the corticospinal excitability changes induced by subsequent deafferentation of the hand [17]. This observation that corticospinal facilitation was greater when pain was present prior to deafferentation, shows that nociceptive input can influence the plasticity induced by a deafferentation, which could be a contributing factor in the cortical somatosensory reorganization observed in chronic pain populations [31, 35, 36].

The aim of the present study was to test whether pain also impacts on the deafferentation-induced plasticity in the somatosensory pathways. Transient ischemic deafferentation of the hand, induced through inflation of a cuff at wrist level, was used as an experimental model of amputation. Somatosensory evoked potentials (SSEPs) associated to the electrical stimulation of the ulnar nerve above the block level were recorded to assess deafferentation-induced somatosensory plasticity, depending on whether pain was already present (Pain condition) or not (No Pain condition). Importantly, nerve stimulations were applied above the cuff used to induce hand deafferentation, so that the changes in SSEPs reflect change in the cortical representation of the deafferented region, rather than the fact that the inflated cuff blocks nerve transmission. This approach aims at characterizing changes occurring directly in the cortical representation of the painful/deafferented body part, rather than looking at adjacent body parts/nerve territory. Reorganization in the cortical representation of adjacent body parts following amputation or transient deafferentation has often been interpreted as occurring “at the expense” of the missing/deafferented body part representation, resulting in reciprocal patterns of change. There is increasing evidence, however, that this is not necessarily the case, as hand amputees appear to retain a representation of their hand, both in the sensory and motor cortex [37–42]. Moreover, it was elegantly shown that increased response of biceps to transcranial magnetic stimulation during distal ischemia induced by an inflated cuff on the forearm is not accompanied by a corresponding decrease in the efferent neural potential recorded in the median and ulnar nerve at a level above the block. This shows the need for studies looking at sensorimotor changes induced in the cortical representation of the deafferented body part itself [43].

## Experimental procedures

### Participants

Fifteen healthy participants (4 males; 3 left-handed; 24.7 years ±4.4) were recruited from Laval University mailing lists for this study. Exclusion criteria were a history of neurological or psychiatric disorders, of musculoskeletal injury affecting the upper limbs, or of chronic pain. Participants were also excluded if they reported any type of acute pain on the day of testing. Each participant provided her/his written informed consent in accordance with the Declaration of Helsinki. The study was approved by the local ethics committee (CER-2009-173, Institut de réadaptation en déficience physique de Québec). Participants received a reimbursement of their travel fees for each visit.

## Experimental design

Each participant completed two experimental sessions, each session corresponding to one of two experimental conditions (*No Pain*, *Pain*) presented in a counterbalanced manner, one to two weeks apart. The two sessions were exactly similar, except that capsaicin cream was applied to the dorsum of the right hand prior to the application of the block in the Pain session, as the experimental model of “pre-amputation pain”. Participants sat comfortably in a chair while keeping their eyes fixed on a static reference situated approximately 1.5 m away, at eye level, to ensure that eye movements were kept to a minimum. Both arms were placed on custom-made adjustable armrests with the forearm pronated, and the fingers slightly flexed to ensure maximum comfort and minimize arm movement during the procedure. Prior to the beginning of the experiment, an EEG sensor net was installed, the stimulation electrodes were positioned bilaterally over the ulnar nerves, just above the medial condyles, and the stimulation thresholds were determined (see details below).

**Fig 1:**
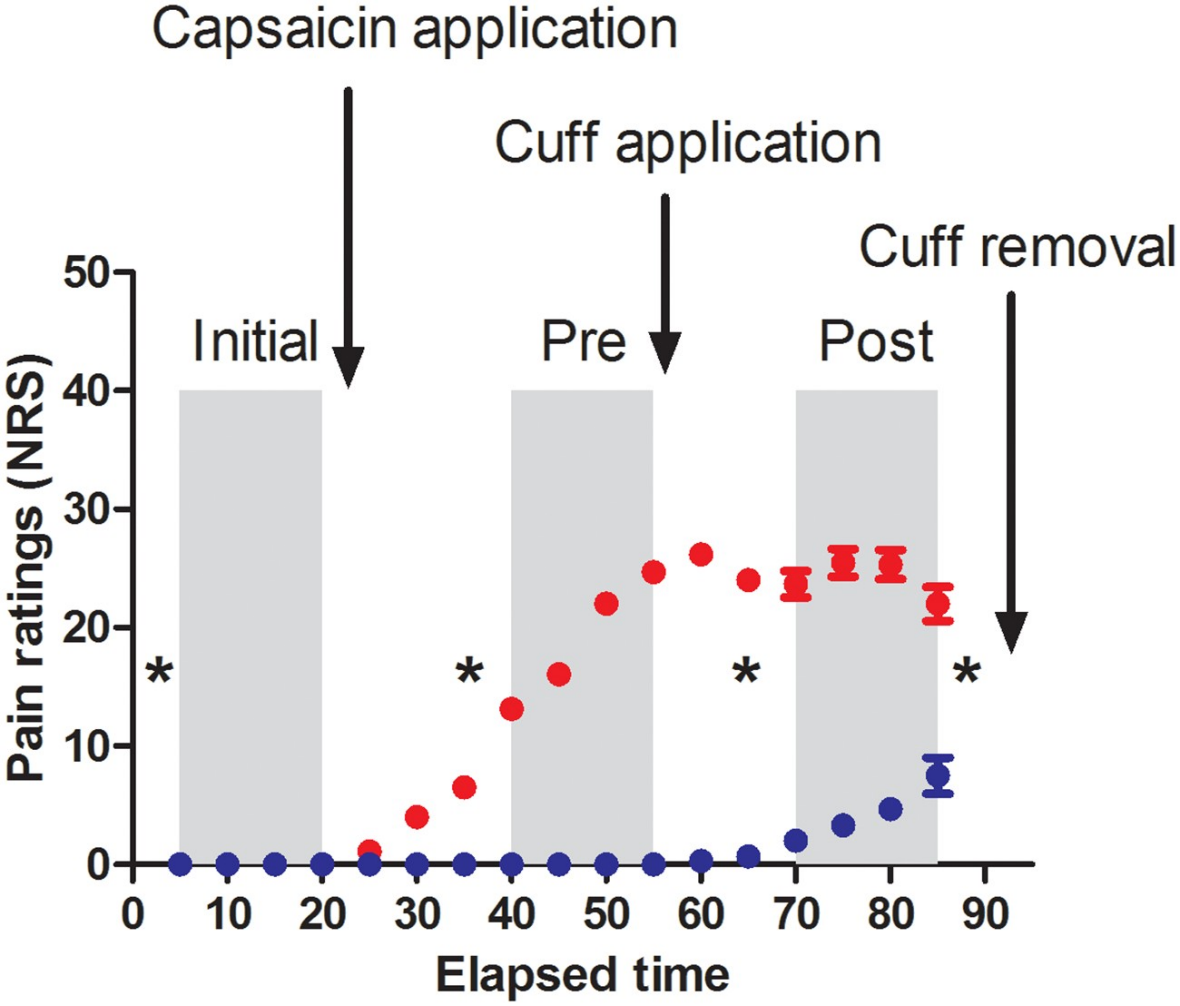
Time course of an experimental session, with the specific timing for each SSEP block (shaded area; three measurement times) with respect to capsaicin application (Pain session only) and the cuff inflation (Pain and No Pain sessions). Red and blue symbols represent the mean pain rating on a numerical rating scale (NRS, /100) in the Pain and No Pain sessions, respectively. Error bars represent the standard error of the mean. Mechanical detection thresholds were assessed just prior to each SSEP block (T0, T35, T65) as well as after the last block (T85) (indicated by asterisks). Then the cuff was removed (T90).

Fig 1 illustrates the experimental design, that was very similar to the one reported in our previous study [17]. In both experimental sessions, temporary ischemic deafferentation was induced through the inflation of a pediatric blood pressure cuff applied just proximal to the right wrist (pressure of 220 mmHg) from T55 to T90 (time in minutes, T0 corresponding to the beginning of the experimental protocol). Deafferentation was monitored using Von Frey hair testing (VFHT). VFHT was performed two times prior to block application (T0 and T35) and two times after (T65 and T85). In the Pain session only, the subjects also received a topical application of 1% capsaicin cream (layer of ~1 mm, over a surface of ~25 cm^2^) on the dorsum of the right hand at T20, i.e. ~30 minutes prior to block application for pain to reach a stable level prior block application. In both sessions, participants were required to provide pain ratings every 5 minutes on a numerical rating scale (NRS), 0 corresponding to No pain and 100 to the Worst pain imaginable. SSEPs were recorded at 3 time periods, starting at T5 (prior to any experimental manipulation), T40 (after pain induction, if applicable, but prior to block inflation) and T70 minutes (after block inflation). Although experimental manipulations (capsaicin and ischemic deafferentation) were applied solely on the right arm, SSEPs were recorded for both right and left arm stimulation, to verify whether the effects were specific to the side exposed to the experimental conditions.

### Electrical stimulation

SSEPs were evoked by electrical stimulation of the right and left ulnar nerves at the elbow, just above the medial condyles, using a square wave pulse of 200 μs (GRASS S88 stimulator with SIU5 stimulus isolation unit; West Warwick, Rhode Island, USA). Intensity was individually adjusted to a comfortable level (110% of the radiating threshold, which was defined as the lowest stimulation intensity required to evoke a clear paresthesia radiating in the fifth digit). Stimulation intensity was adjusted separately for each side and was kept constant throughout all SSEP blocks. For each SSEP block, 6 trains of stimuli with a duration of 120 seconds were applied at 1Hz (120 stimuli / train). Three trains were performed on each side, alternating between sides (starting with the right side), for a total of 360 stimuli for each side at each SSEPs time point of the experiment.

### EEG recordings

Electroencephalographic (EEG) data was acquired with a 128-channel system consisting in a saline-soaked HydroCel Geodesic Sensor Net fitted on the scalp according to the International 10–10 System, a Net Amps 300 amplifier and the Net Station software (Electrical Geodesics Inc., Eugene, OR, USA). Data was sampled at 1000 Hz and referenced to Fz. Scalp impedance for each electrode was monitored before each recording block and kept below 50 kΩ. Time-locked EEG data (synchronized to the electrical stimulation) was acquired through Net Station, converted to binary RAW data, and then imported to Brainstorm [44] for further analyses.

### Mechanical detection thresholds

Testing was performed by perpendicularly applying von Frey monofilaments (Stoelting, Wood Dale, IL, USA) to the glabrous skin on the ulnar side of the hand until it bows. The number of the monofilament represents the logarithm of 10-times the force required in milligrams required to bow it. Testing started with filament 2.83, applied three times with an inter-stimuli interval varying between 2 and 15 seconds (to minimize the predictability of each stimulus). Participants were instructed to give a verbal response whenever a stimulus was felt. A method of limits with ascending and descending monofilament numbers was used to identify the mechanical detection threshold, defined as the smallest monofilament for which three consecutive positive responses were obtained [45].

## Data analysis

**Fig 2:**
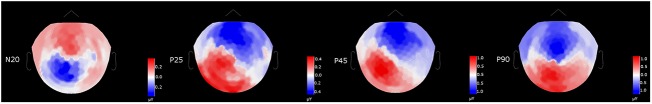
Mean scalp topography during of the different components of the SSEPs extracted at 15 ms (N20), 19 ms (P25), 43 ms (P45) and 84 ms (P90). Note that these maps are based solely on the first block of SSEPs, i.e. prior to any experimental manipulation.

Before extracting single trial epochs, continuous EEG RAW data was visually inspected for artifacts (blinks, electromyographic activity, and so forth) and time segments containing artifacts were removed prior to conducting further analyses. Importantly, pre-processing was performed by an investigator blinded to the experimental conditions during which the signal was acquired. EEG single trial epochs were extracted from 100 ms before to 200 ms after the electrical stimulation and individual electrodes for each epoch were baseline corrected using a window between -100 ms and -10 ms (with respect to the onset of the electrical stimulation). Single epochs from a given time point and condition were then averaged, and visual inspection of each electrode was performed to detect potential outliers. The electrode of interest for each component of the SSEPs was determined by examining the centro-parietal region, the parietal region and the region located at the edge of the centro-parietal and the temporal cortex on the mean scalp topographies obtained during the first block of SSEPS, i.e. prior to any experimental manipulation (see Fig 2). The selected electrodes were CP3 for the left hemisphere (right hand stimulation) and CP4 for the right hemisphere during left hand stimulation.

The SSEPs of the first block (i.e. prior to any experimental manipulation) were inspected for each participant to determine the onset of the first negative deflection (corresponding to the N20 wave [46]), immediately followed by a positive deflection (corresponding to the P25 wave). The P45 (37–47 ms) and P90 (82–112 ms) components were also located. The average latency across all participants was used to extract the amplitudes of each component. Single peak amplitudes were extracted for the N20 (15 ms) and the P25 (19 ms) waves, whereas average amplitude within a time window from -5 ms to +5 ms relative to each identified component was extracted (P45: 38 ms to 48 ms; P90: 79 ms to 89 ms). Note that the peak latencies are shorter than what is typically described in the literature given the fact that the stimulation was applied at the elbow level, while they are typically applied at wrist level. To control for SSEPs variation across sessions, the average amplitude during the first block of stimulation was subtracted from the amplitude obtained during the second and third blocks (pre-inflation and post-inflation). All statistical analyses were performed on these normalized amplitudes.

## Statistical analysis

As in our previous study using a similar paradigm [17], two-way repeated-measure analyses of variance (ANOVA) were performed to assess the effect of Condition (Pain/No Pain) and Time (Pre-inflation/Post-inflation) on SSEPs. Independent analyses were performed for each side (right/left stimulation) and each component of interest (N20, P25, P45 and P90). A main effect of the Condition can be interpreted as the direct effect of pain on SSEP, a main effect of Time as the effect of the temporary deafferentation, while an interaction is interpreted as an effect of pain on the plasticity induced by the temporary ischemic deafferentation, reflecting state-dependent plasticity. Partial ETA squared (η^2^_p_) and partial omega squared (ω_p_^2^) were also calculated for each significant effect. Partial ETA squared was used as it is the most commonly reported effect size while partial omega squared is the most representative of the population effect size [47]. Prior to this analysis, it was first verified that the order of the sessions did not influence the SSEP amplitude (N20: F(1,13) = 1.527, p = 0.435; P25: F(1,13) = 0.095, p = 0.372; P45: F(1,13) = 2.868, p = 0.153; P90: F(1,13) = 0.003, p = 0.652). Therefore, the order of the session was not entered in the ANOVA model.

A similar two-way repeated-measure ANOVA was performed on mechanical detection thresholds. Finally, an ANOVA was performed to verify that there was no difference between Conditions (Pain/No Pain) and Sides (Right/Left) on the average intensity of the individually adjusted stimulation intensity. All statistical analyses were performed using SPSS 21 software (SPSS Inc., Chicago, IL, USA) and statistical significance was set at p < 0.05. P-values between 0.05 and 0.06 were considered as trend for a difference.

## Results

### Mechanical detection thresholds

**Fig 3:**
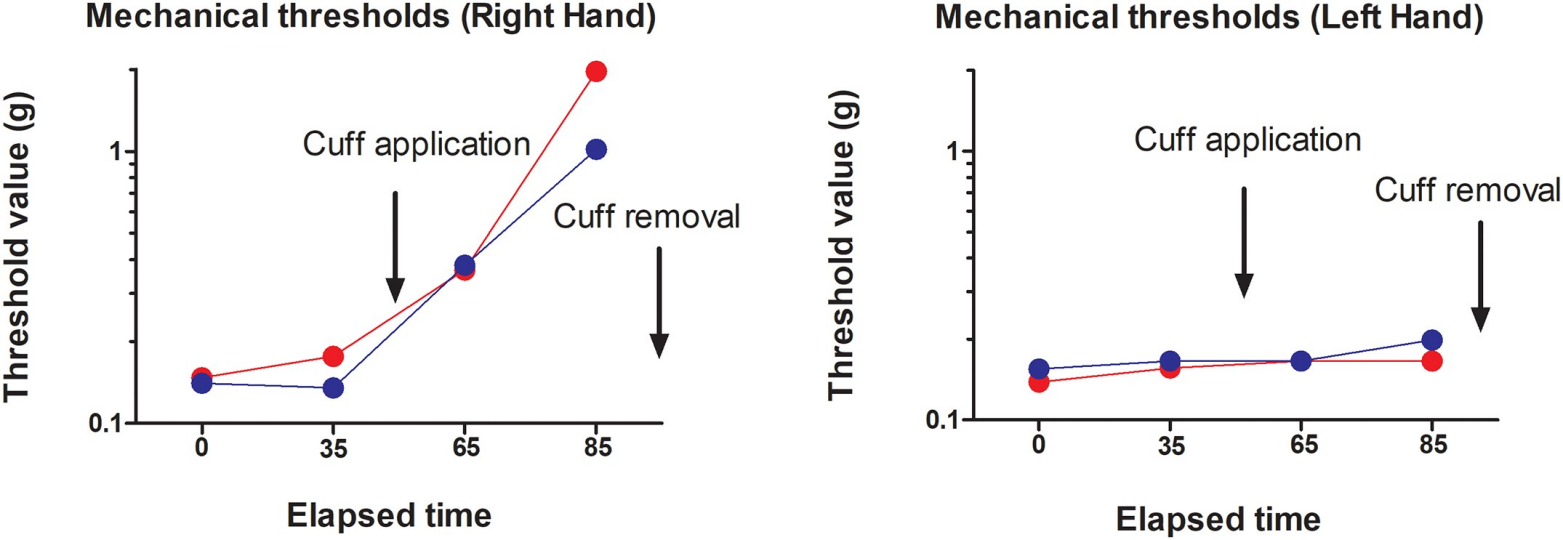
Mean mechanical detection threshold (grams) for each measure throughout each session. Red and blue symbols represent the Pain and the No Pain sessions, respectively.

Fig 3 shows the mechanical detection thresholds, over time, for each hand. A significant increase in mechanical detection thresholds was observed over time for the right hand only (Mean ± SD: Pre-inflation: 3.17 ± 0.29; Post-inflation: 4.00 ± 0.29; Pain: 4.29 ± 0.73; No Pain: 3.17 ± 0.20)) F_(3,39)_ = 76.4, p < 0.001, η^2^_p_ = 0.86), confirming that the inflated cuff successfully induced gradual deafferentation. No effect of Condition or Condition X Time interaction was observed.

### Somatosensory evoked potentials

The average intensity of the individually adjusted stimulation intensity was 82.55 V ± 20.54, and did not differ between left and right sides (F(1,13) = 0.27, p = 0.61) nor between experimental conditions (F(1,13) = 0.22, p = 0.65).

**Fig 4:**
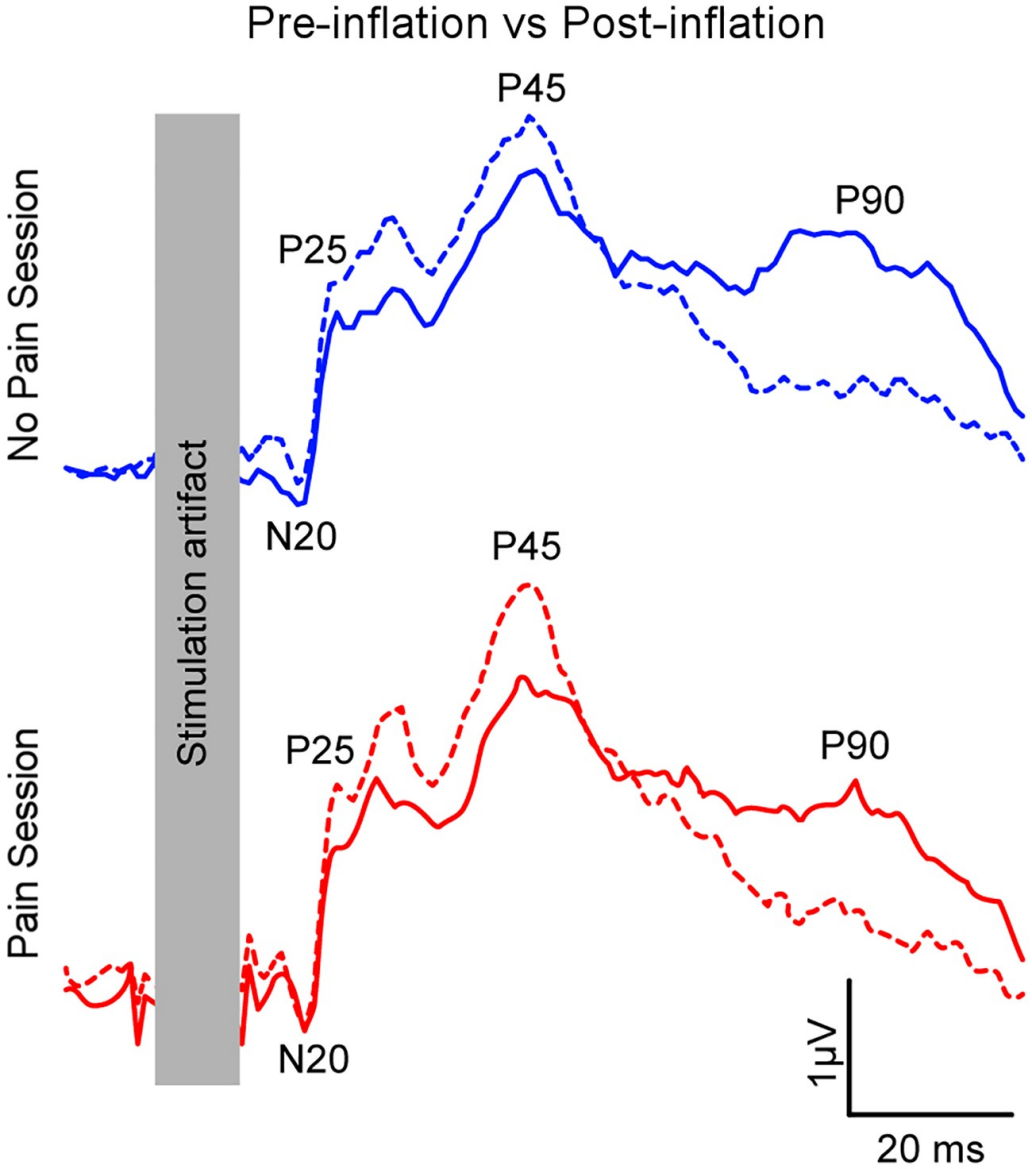
Mean curve of SSEPs recorded from CP3 (left hemisphere) at the Pre-inflation period (dashed line) vs. the Post-inflation period (full line) in each condition. Blue lines represent the No Pain session and red lines represent the Pain session. Note that for comparisons between sessions, data was normalized against the initial block of stimulation. Normalized amplitude of SSEP components used for statistical analyses is presented in Fig 5.

**Fig 5:**
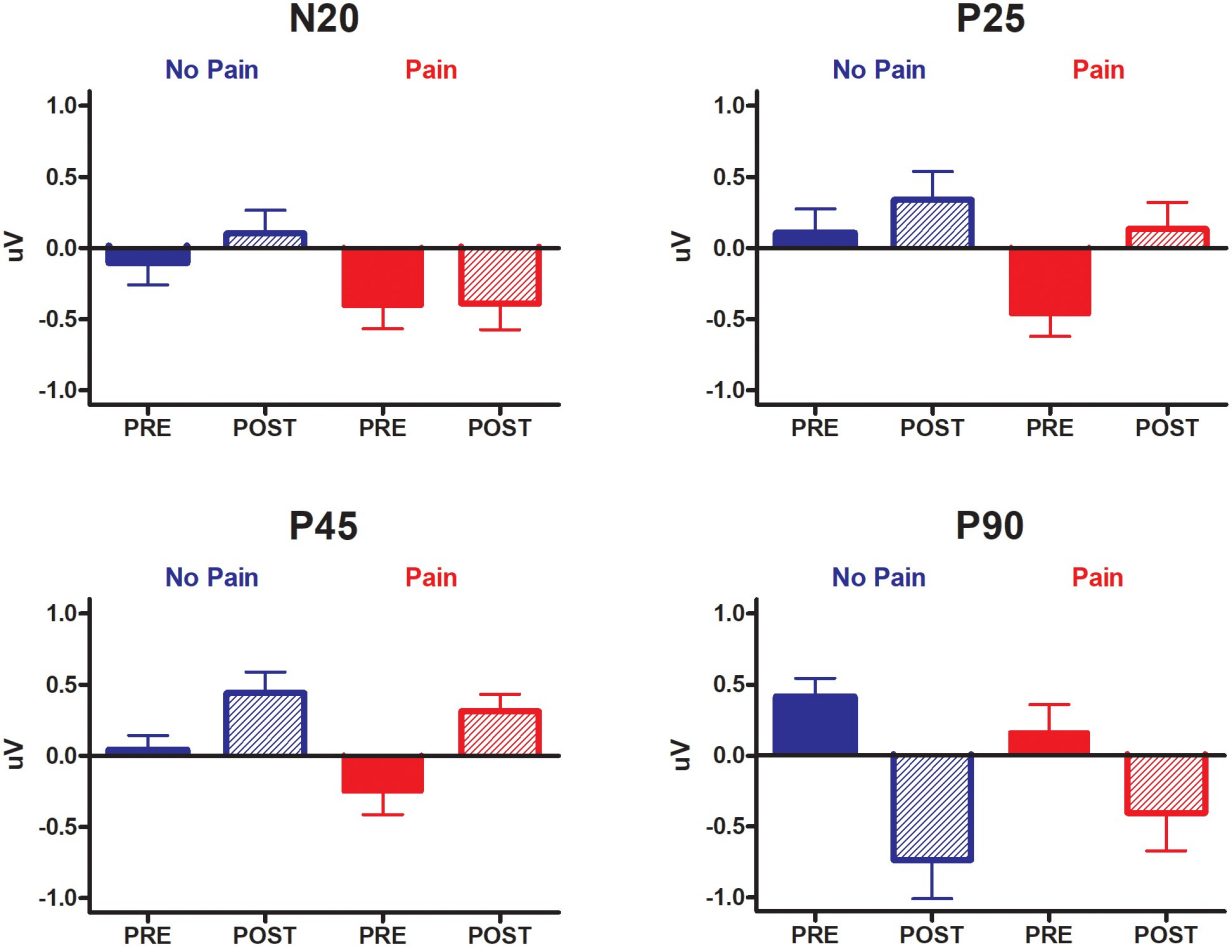
Mean normalized amplitude of each SSEP component of interest recorded from CP3 (left hemisphere) for Pre-inflation (filled columns) and Post-inflation (dashed columns) periods, in the Pain (red) and No Pain (blue) sessions. Error bars represent SEM.

SSEPs waveforms recorded at different time points and conditions are presented in Fig 4, and the results extracted for each component are presented in Fig 5 (left hemisphere only, i.e. contralateral to the limb to which experimental conditions were applied). Statistical results for each SSEP’s component on each side are summarized in Table 1.

**Table 1:** List of all the significant effects (or trends) on the amplitudes of each component at their respective electrodes of interest (CP3 for the right limb and CP4 for the left limb).

		*Condition* *(Pain/No Pain)*			*Time* *(Pre/Post-inflation)*				Interaction Condition X Time				
Component	Electrode	F_(1,14)_	p	η^2^_p_	ω_p_^2^	F_(1,14)_	p	η^2^_p_	ω_p_^2^	F_(1,14)_	p	η^2^_p_	ω_p_^2^
N20	CP3	5.62	.033	0.29	0.25	-	-	-	-	-	-	-	-
	CP4	-	-	-	-	-	-	-	-	-	-	-	-
P25	CP3	4.21	.059	0.23	0.19	8.19	.013	0.37	0.31	-	-	-	-
	CP4	-	-	-	-	-	-	-	-	-	-	-	-
P45	CP3	-	-	-	-	11.01	.005	0.44	0.42	-	-	-	-
	CP4	-	-	-	-	4.56	.051	0.25	0.20	-	-	-	-
P90	CP3	-	-	-	-	14.17	.002	0.50	0.48	4.64	.049	0.25	0.21
	CP4	-	-	-	-	-	-	-	-	-	-	-	-

η^2^_p_: Partial ETA squared which corresponds to an observed effect for each condition.

ω_p_^2^: Partial omega squared which corresponds to an observed effect for each condition.

#### SSEPs for the right limb (left hemisphere)

The presence of hand pain (main effect of Condition) resulted in a significant reduction in the N20 amplitude (p = 0.033) and a trend for decrease in the P25 amplitude (p = 0.059).

Temporary ischemic deafferentation (main effect of Time) caused a significant increase in the P25 amplitude (p = 0.013) and the P45 amplitude (p = 0.005), together with a significant reduction of the P90 amplitude (p = 0.002).

Finally, a significant interaction was observed only for the P90 amplitude (p = 0.049), revealing that the reduction of P90 amplitude caused by the deafferentation was attenuated by the presence of hand pain.

#### SSEPs for the left limb (right hemisphere)

No significant effect of Condition, Time or Condition X Time interaction or SSEPs was observed for the left limb, apart from a trend for an increase of the P45 during transient deafferentation (p = 0.051). However, the observed effect size was smaller than for the right hemisphere (η^2^_p_ = 025 vs. 0.44, respectively).

## Discussion

The aim of this study was to test whether pain impacts the deafferentation-induced plasticity in the somatosensory pathways. Significant effects were observed only for SSEP for the right limb, indicating that the effects of the experimental conditions were specific to the side exposed to those manipulations. The significant Time x Condition interaction found for the P90 confirms the presence of such state-dependent plasticity, nociceptive input decreasing the reduction observed in response to deafferentation. However, before elaborating on this interaction between the effect of pain and that of transient deafferentation, the independent effects of pain and transient deafferentation will be discussed.

Pain alone was found to influence early components of SSEPs (N20, P25) evoked from the painful hand (with no effect on the contralateral side). This observation is consistent with previous studies assessing median nerve SSEPs following capsaicin application [48, 49]. Interestingly, one of these studies showed a decrease in SSEPs during a local, but not remote, application of capsaicin [49], which is consistent with the lack of contralateral effect. A similar pain-induced depression of the P25 component of the ulnar nerve SSEPs was also reported after injection of a Levo-Ascorbic (L-AS) solution in the first dorsal interosseous muscle [50], suggesting that this effect is not modality-specific.

Transient ischemic deafferentation induced more complex effects. Early components (P25, P45) were increased by the deafferentation of the territory of the stimulated nerve, consistent with previous observations for deafferentation of adjacent body parts [51–54]. Conversely, the P90 was reduced. This pattern of results suggests independent effects on SI and SII. Indeed, results from MEG studies indicate that the earliest components of the SEP originate in areas 3b and 1 within SI, while later SEP components originate from SII [55–57]. Reduction of the P90 component after deafferentation might play a role in non-painful phantom sensations. For instance, perception of a supernumerary limb has been found to be associated with the suppression of activity in contralateral SII, suggesting that SII play a role in the maintenance of the body schema [58].

This effect of deafferentation on the P90 was partially blocked by the presence of pain (although pain *per se* had no effect on that component), confirming that pain can modulate the plasticity induced by another event (e.g. deafferentation). It is interesting to observe the presence of an interaction only for P90, given that SII was identified as an integration area of nociceptive (N140–P170 CO2 laser-evoked potentials) and non-nociceptive (N60–P90 electrical evoked potentials) somatosensory inputs [59]. Our previous study using a similar paradigm to investigate corticospinal changes showed that deafferentation-induced changes were primed (rather than blocked) by the presence of nociceptive input prior to the deafferentation [17]. This discrepancy might be explained either by the fact that in the transcranial magnetic study, the muscles above the block were tested (i.e. body part adjacent to the deafferented area) while in the present study SSEPs were recorded by stimulating a nerve with a territory corresponding to the deafferented body part itself. It might alternatively reflect the fact that nociceptive input has a different impact on plasticity occurring in the motor and in the sensory areas. The complexity of the different functional contexts (sensory versus sensorimotor) and different methods used to assess cortical reorganization, as well as the various sources of plasticity following amputation, have been highlighted in a recent review paper [32].

How can these results contribute to our understanding of PLP? Interestingly, it has recently been proposed that cortical changes occurring after an amputation are due to a combination of loss of sensory inputs and pain experience [33]. According to these authors, sensory deprivation would result in disrupted local cortical representations, while pain would contribute to maintain local cortical representations and disrupt inter-regional connectivity. Our results, showing that some of the effects of deafferentation are partially blocked by the presence of pain, are consistent with this hypothesis. An interesting future direction would be to develop an fMRI paradigm using the capsaicin and transient ischemic deafferentation model employed here to allow more direct comparisons with the measures employed in the amputee population (typically focusing on remapping of hand vs. face representation). Another interesting question would be to determine whether capsaicin would also impact on the cortical reorganization caused by limb immobilization [60], which would contribute to explain the cortical somatosensory reorganization observed in chronic pain populations beyond PLP.

Some limitations of the present study need to be highlighted. First, temporary ischemic deafferentation is obviously an imperfect model of amputation-induced reorganization, although it can offer some insight into these complex interactions, by allowing the experimental manipulation of various variables. One of these limitations is the fact that temporary ischemic deafferentation itself results in some pain (see Fig 1). Second, multiple ANOVAs (2 sides x 4 SSEP components were performed, without correcting p-values. We considered it overly conservative to apply a correction between ANOVAs. Importantly, large effect sizes (η^2^_p_ ≥ 0.25; ω_p_^2^ ≥ 0.19) were found for each statistically significant result, which increased the level of confidence in the observed effect. Finally, the sample size was limited and not representative of the general population (mainly composed of female university students), which decreases the generalizability of the results. Nevertheless, the pattern of results observed for the isolated effect of pain and of temporary deafferentation were very consistent with previous reports in literature.

In conclusion, results of the present study show that the presence of nociceptive input prior to a deafferentation can modulate the plasticity that it induces. This highlights the potential importance of pain in explaining some of the variability in outcomes after injury, as differences in reorganization occurring in somatosensory areas might in turn contribute to differences in painful or nonpainful (e.g. altered body perception) sensations in clinical populations with deafferentation.

## Supporting information

S1 Table Mean normalized amplitude of each SSEP component of interest recorded from CP3 or CP4. S1A: N20 S1B: P25 S1C: P45 S1D: P90.
(PDF)
