# Supplementary material for: Effect of pain on deafferentation-induced modulation of somatosensory evoked potentials
Source: PLoS One. 2018 Oct 22;13(10):e0206141. doi: 10.1371/journal.pone.0206141 (PMC6197665; doi:10.1371/journal.pone.0206141)
Supplement: S1 Table — S1A: N20 S1B: P25 S1C: P45 S1D: P90. (PDF) [file pone.0206141.s001.pdf]

S1 A-D

S1A

	N20							
	Right arm stimulation (CP3)				Left arm stimulation (CP4)			
	No pain		Pain		No pain		Pain	
Subject	Pre-inflation	Post-Inflation	Pre-inflation	Post-Inflation	Pre-inflation	Post-Inflation	Pre-inflation	Post-Inflation
1	-0.323	0.842	-1.126	-1.257	-0.654	-0.915	-1.345	-2.221
2	0.084	0.139	-0.143	-0.617	-0.527	-2.308	-1.352	-0.644
3	-0.402	-0.498	0.269	0.208	1.669	-0.798	-0.381	-0.692
4	-0.038	0.495	-0.222	-0.354	-0.043	1.075	0.602	-0.287
5	-0.333	-0.253	-0.429	-1.025	0.151	1.058	0.286	0.761
6	-1.412	-0.879	-1.530	-1.627	1.134	-2.027	2.030	1.809
7	0.021	-0.087	-0.615	-0.816	-0.360	0.175	-0.912	-0.539
8	-0.666	-0.062	-0.001	0.654	-0.515	-1.601	-1.529	-1.351
9	-0.646	-0.663	-1.329	-1.019	0.240	1.010	0.562	1.223
10	0.269	0.212	-0.783	0.081	-0.001	-0.155	-0.422	-0.583
11	-0.565	0.199	0.627	0.605	-0.538	-0.461	-1.384	-0.380
12	0.907	0.429	0.290	-0.206	-0.498	0.092	-1.285	-1.682
13	0.783	1.508	-0.674	0.408	0.031	-0.588	-0.264	-0.459
14	0.473	0.618	0.041	0.083	0.086	-0.163	0.399	0.668
15	0.352	-0.437	-0.474	-0.949	0.020	0.120	-0.494	-0.316
Mean	-0.100	0.104	-0.406	-0.389	0.013	-0.366	-0.366	-0.313
SD	0.613	0.624	0.619	0.716	0.643	1.050	1.001	1.068

S1B

	P25							
	Right arm stimulation (CP3)				Left arm stimulation (CP4)			
	No pain		Pain		No pain		Pain	
Subject	Pre-inflation	Post-Inflation	Pre-inflation	Post-Inflation	Pre-inflation	Post-Inflation	Pre-inflation	Post-Inflation
1	0.001	1.145	-1.468	0.599	0.669	1.681	-0.855	-1.095
2	-0.109	0.539	-0.285	-0.285	-0.270	-0.159	-1.009	-0.277
3	0.308	1.665	-0.265	-0.528	-1.576	0.917	0.409	-0.337
4	-1.082	-0.658	-0.410	-0.574	-0.287	-0.852	0.147	1.078
5	0.397	0.157	-1.297	1.868	-0.075	-0.291	2.480	-0.594
6	-0.754	-0.764	-1.334	-0.082	0.276	-2.985	1.968	1.267
7	0.048	-0.273	-0.588	-1.038	-0.013	0.196	-0.937	-0.630
8	-0.694	-0.163	-0.185	0.041	0.404	-0.231	0.485	0.332
9	-0.155	-0.455	-0.495	-0.168	0.416	0.808	0.437	1.257
10	1.163	1.066	-0.961	-0.162	-0.905	-0.754	-0.738	-1.345
11	-0.428	-0.401	0.295	0.723	1.243	0.973	0.087	-0.256
12	0.694	0.500	0.503	1.048	-0.871	0.435	-0.329	-1.055
13	0.831	1.424	-0.265	0.577	0.006	-0.786	-0.001	0.062
14	0.235	0.610	-0.624	0.087	-0.022	0.152	-0.052	0.436
15	1.064	0.674	0.448	-0.092	-0.045	-0.595	-0.220	0.945
Mean	0.101	0.338	-0.462	0.134	-0.070	-0.099	0.125	-0.014
SD	0.668	0.775	0.614	0.726	0.684	1.092	0.991	0.873

S1C

	P45							
	Right arm stimulation (CP3)				Left arm stimulation (CP4)			
	No pain		Pain		No pain		Pain	
Subject	Pre-inflation	Post-Inflation	Pre-inflation	Post-Inflation	Pre-inflation	Post-Inflation	Pre-inflation	Post-Inflation
1	-0.157	12.113	-15.946	15.007	11.435	17.598	3.617	-3.390
2	2.940	5.406	7.544	3.374	2.318	22.894	-2.783	13.630
3	8.531	15.975	-12.044	1.881	-23.183	5.749	-4.680	5.540
4	-3.388	-4.038	-3.141	0.115	3.211	5.249	-4.103	-2.910
5	-0.612	6.459	-3.748	4.702	-2.564	-8.619	3.162	-5.960
6	-1.159	6.133	-10.873	2.724	-33.046	-7.778	1.385	-2.020
7	3.831	9.640	3.231	-4.445	6.074	-7.330	-6.722	-5.530
8	-5.356	9.691	5.846	9.416	0.645	1.810	20.301	20.740
9	-5.208	-7.475	1.437	1.974	1.778	11.338	5.451	3.520
10	4.342	6.620	-8.786	-1.038	-10.913	-9.193	-0.457	-0.170
11	-5.197	3.960	-2.160	3.940	2.508	14.533	-4.335	5.390
12	0.474	1.358	-1.816	4.575	-7.865	3.145	7.367	3.040
13	-3.011	1.977	-5.741	-1.124	0.065	1.083	-1.044	3.430
14	6.874	8.406	2.729	-0.672	0.585	0.577	-3.404	-4.210
15	3.337	-3.584	1.899	11.096	4.719	4.956	0.712	12.170
Mean	0.416	4.843	-2.771	3.435	-2.949	3.734	0.964	2.885
SD	4.441	6.361	6.861	5.123	11.657	9.773	6.714	7.747

S1D

	P90							
	Right arm stimulation (CP3)				Left arm stimulation (CP4)			
	No pain		Pain		No pain		Pain	
Subject	Pre-inflation	Post-Inflation	Pre-inflation	Post-Inflation	Pre-inflation	Post-Inflation	Pre-inflation	Post-Inflation
1	8.615	-32.513	0.689	-4.259	-25.614	-29.694	-20.710	-39.914
2	1.722	-17.561	22.098	8.233	13.771	59.779	-4.593	-9.934
3	9.384	-7.559	6.782	12.793	0.803	7.721	7.478	9.295
4	2.613	-3.977	-6.636	-3.495	-10.899	-7.109	-5.592	-1.170
5	9.929	-27.594	1.445	-32.708	0.934	-4.201	-22.840	-9.343
6	11.875	11.519	-9.046	-14.775	-20.150	-9.024	-15.446	2.472
7	5.353	2.136	11.080	7.660	3.602	0.549	-26.307	-10.110
8	4.513	-5.552	2.121	-6.264	-0.914	7.639	0.038	-23.560
9	11.591	-4.917	0.319	-2.674	-5.589	-12.414	-7.113	-5.766
10	-2.688	3.023	-5.491	-5.310	-3.775	-3.867	26.518	19.050
11	1.047	-4.266	4.661	3.685	6.653	2.739	-4.371	1.980
12	-5.185	-16.478	-4.991	-8.243	-14.406	-0.476	18.928	8.008
13	1.584	0.805	-7.373	-16.657	6.122	18.563	2.989	3.916
14	10.270	-7.108	9.883	0.912	5.204	4.719	2.812	-4.981
15	-2.748	-11.420	1.404	-5.709	3.248	-3.119	0.804	3.489
Mean	4.525	-8.098	1.796	-4.454	-2.734	2.120	-3.160	-3.771
SD	5.598	11.616	8.334	11.269	10.844	19.289	14.610	14.226
